# Virtual Sensing and Sensors Selection for Efficient Temperature Monitoring in Indoor Environments †

**DOI:** 10.3390/s21082728

**Published:** 2021-04-13

**Authors:** Andrea Brunello, Andrea Urgolo, Federico Pittino, András Montvay, Angelo Montanari

**Affiliations:** 1 Data Science and Automatic Verification Laboratory, University of Udine, Via delle Scienze 206, 33100 Udine, Italy; angelo.montanari@uniud.it; 2 Silicon Austria Labs GmBH, Europastraße 12, A-9524 Villach, Austria; federico.pittino@silicon-austria.com (F.P.); andras.montvay@silicon-austria.com (A.M.)

**Keywords:** virtual sensing, sensor selection, temperature monitoring, machine learning, neural networks, particle filters, distance metrics

## Abstract

Real-time estimation of temperatures in indoor environments is critical for several reasons, including the upkeep of comfort levels, the fulfillment of legal requirements, and energy efficiency. Unfortunately, setting an adequate number of sensors at the desired locations to ensure a uniform monitoring of the temperature in a given premise may be troublesome. Virtual sensing is a set of techniques to replace a subset of physical sensors by virtual ones, allowing the monitoring of unreachable locations, reducing the sensors deployment costs, and providing a fallback solution for sensor failures. In this paper, we deal with temperature monitoring in an open space office, where a set of physical sensors is deployed at uneven locations. Our main goal is to develop a black-box virtual sensing framework, completely independent of the physical characteristics of the considered scenario, that, in principle, can be adapted to any indoor environment. We first perform a systematic analysis of various distance metrics that can be used to determine the best sensors on which to base temperature monitoring. Then, following a genetic programming approach, we design a novel metric that combines and summarizes information brought by the considered distance metrics, outperforming their effectiveness. Thereafter, we propose a general and automatic approach to the problem of determining the best subset of sensors that are worth keeping in a given room. Leveraging the selected sensors, we then conduct a comprehensive assessment of different strategies for the prediction of temperatures observed by physical sensors based on other sensors’ data, also evaluating the reliability of the generated outputs. The results show that, at least in the given scenario, the proposed black-box approach is capable of automatically selecting a subset of sensors and of deriving a virtual sensing model for an accurate and efficient monitoring of the environment.

## 1. Introduction

Sensing means to grasp the nature, significance, or meaning of a physical phenomenon or stimulus. A sensor can be defined as a device which measures a physical quantity and transforms it into a signal that can be interpreted by an instrument or an observer. Sensors can be partitioned into two main classes: physical sensors, which measure physical phenomena directly, and virtual sensors, which are designed to process various physically measured data and to produce values approximating those that would be directly reported by physical sensors [1].

Virtual sensing relies on data captured by physical sensors and on the use of suitable models to combine them in order to estimate a difficult or expensive-to-measure quantity. Limitations may indeed be present regarding where actual sensors may be placed, and they can be either of physical or of economical nature. For this reason, over the past decades, virtual sensors have been successfully developed and adopted in various fields, exploiting any indirect approach that relies on the outputs of other physical or virtual sensors, process models, and property relations [2].

One of the many applications of virtual sensing is connected to the accurate measurement of temperature in an indoor environment. Such a scenario comprises a large variety of applications, ranging from control of the comfort and energy efficiency in an office or residential space to the monitoring of safe temperature levels in an industrial environment. To obtain an accurate temperature measurement, a very large number of sensors typically need to be deployed in the environment, covering all possible heat sources and sinks, and monitoring heat propagation. Virtual sensing is a promising technique for decreasing the complexity of the network of monitoring physical sensors by replacing some of them with virtual ones, in which the temperature value is inferred from the physical measurements, while retaining measurement accuracy and fine spatio-temporal granularity. This technique, however, poses several challenges, namely how to choose the location of the physical sensors, how many of them are needed, and finding the best prediction method for optimal accuracy. In this paper, we address these issues, taking the temperature monitoring in an open space office at Silicon Austria Labs premise in Villach (Austria) as a case study.

The approach we developed includes, as a first step, the selection of a subset of sensors on which the prediction is based. Such a selection is made by evaluating several distance metrics, including a novel metric obtained by a combination of existing ones through a genetic programming approach. The proposed solution is actually a black-box one—that is, it is designed with the idea of making it easily generalizable to any other indoor environment. To achieve this, the presence of elements in the room capable of influencing the internal temperature, like windows and radiators, was not modeled directly, but through the automatic selection of sensors adjacent to them, which should carry useful information for the task of temperature prediction. Afterwards, based on the developed metric and on a set of thoroughly engineered features, we performed a comprehensive evaluation of several temperature prediction techniques, which include particle filters, machine learning (in particular, linear regression and ensembles), and deep learning solutions. In order to obtain an indication of the reliability of the generated predictions, we also carried out a study based on the estimation of confidence intervals. Overall, the devised approach can be considered as an automatic black-box methodology to select the number and position of the sensors to be retained and the ones that will be virtualized.

The paper is organized as follows. Section 2 gives a short account of related work on virtual sensing and sensor selection. Then, Section 3 introduces the considered case study. Next, we turn to the experimental part of the work, which consists of three sections. We begin with Section 4, where we perform a preliminary descriptive analysis of our dataset. Then, in Section 5, the data pre-processing phase is described, along with the feature engineering step, the development of a novel sensor distance metric based on a genetic programming approach, and the proposal of a domain-independent sensor selection technique. On the basis of the results of the pre-processing tasks, Section 6 thoroughly describes the temperature prediction experiments, and illustrates the main results, including a study of the prediction’s reliability. In addition, a comparison with respect to the performance achievable by means of a brute force sensor selection approach is carried out. Section 7 concludes the main body of the paper with a summary of the main take-home messages and an outline of a high-level procedure to replicate the proposed approach in any indoor environment. Finally, Section 8 provides an assessment of the work done and outlines directions for future work.

## 2. Related Work

The present work studies the problems of virtual sensing and sensor subset selection. Accordingly, we start the analysis of related work with a brief introduction to virtual sensing by analyzing some of the most important approaches proposed in the literature, then we focus on sensor subset selection considering the various application areas where the problem has been addressed.

Over the years, virtual sensing has found application in several domains including robotics, automation, anomaly/leak detection, air quality control, active noise suppression, wireless communication, automotive, and transportation [1,2,3]. In the literature, three main virtual sensing modeling methods have been taken into account: (i) *first-principle*, where virtual sensors are developed by hand on the basis of fundamental laws of physics and an extensive domain knowledge; (ii) *black-box*, where models capable of exploiting empirical correlations present in the data are built, without any knowledge of the underlying physical processes (most of the statistical and machine learning methods belong to this category); (iii) *grey-box*, which exploits a combination of first-principle and black-box approaches. In this work, we focus on the latter two approaches, in order to develop generic models that do not require complex domain knowledge and are not tied to the specific use case.

As for black-box techniques, the estimation of a variable at locations where it has not been observed by exploiting the data available at other locations can be made by means of approaches commonly used for the interpolation of scatter points, such as Inverse Distance Weighting (IDW) and Kriging [4]. IDW assumes that the interpolating surface is more influenced by closer points than distant ones, while Kriging is a geostatistical regression method used in spatial analysis that spatially interpolates a quantity minimizing the mean squared error. As it comes out, both approaches find difficult application in real-world settings, typically being affected by spatial-temporal anisotropy and unable to deal with non-linear relations among predictor variables. When partial observations are made and random perturbations are present in the data, methods such as Kalman filters and particle filters [5] can be relied on for the estimation of the internal states of dynamical systems. These methods compute the unknown quantities through posterior distributions obtained from Bayesian inference models [6,7]. Although particle filters are not subject to the constraints of non-Gaussianity of perturbations and linearity of the dynamic systems that affect Kalman ones, they have some disadvantages as well, related to possible resampling biases and to the coarseness of the definition of the likelihood distribution, which may be unable to capture all relevant real-world characteristics. To overcome the limitations of these techniques, some authors explored approaches based on machine learning, like support vector machines, decision trees, and ensembles (such as random forests) [8,9,10]. More recently, deep learning solutions have been considered as well [11,12,13,14], and were deemed able to exploit implicit information on temporal trends and spatial associations among sensors. As an example, in [15,16] the authors employ a Long Short-Term Memory (LSTM) network and a combined Convolutional LSTM (ConvLSTM) network, respectively, capable of learning from long-term dependencies in spatial-temporal information. Despite the latter ConvLSTM-based model achieving a good performance, it is not applicable in context like ours, where the positions of sensors are fixed and quite sparse.

As for the sensor subset selection problem, different approaches have been explored in the literature in various domains. In [17], an optimal sensor selection and fusion solution is proposed for the case of an heat exchanger fouling diagnosis in aerospace systems. It is based on the minimum Redundancy Maximum Relevance (mRMR) algorithm, and it can be applied only for classification tasks with discretized features. Another sensor selection approach for optimal Fault Detection and Isolation (FDI) tests in complex systems is presented in [18]. In this case, steady-state or dynamic models are assumed to be available, and the estimation is based on their contribution to information gain through Hellinger distance (HD) and Kullback–Leibler divergence (KLD).

In the context of mobile crowd sensing (MCS) systems, which leverage a public crowd equipped with various mobile devices for large-scale sensing tasks, a solution based on reverse combinatorial auctions is proposed in [19], which integrates the concepts of social welfare, quality of information, and cost required by each single user to provide an observation, in order to select an optimal subset of users from whom it is convenient to request data in exchange for a reward. This approach is not applicable to our case, as both the quality level of the sensors and the cost of each observation are the same.

A different class of problems where sensor selection techniques can be exploited is that of sensor scheduling problems, where one or more sensors have to be selected at every time step. As an example, in the domain of linear dynamical systems, a greedy algorithm for sensor scheduling based on submodular error functions, is described in [20]. As another example, in the context of active robotic mapping, a technique to prune the search tree of all possible sensor schedules, based on a weighted function of the error covariances related to the state estimates, is illustrated in [21]. Finally, an algorithm for stochastic sensor selection minimizing the expected error covariance, based on Kalman filters with an underlying Hidden Markov Model, is proposed in [22]; it relies on the assumptions of process model linearity and sensor noise Gaussianity.

Finally, another domain where sensor selection techniques are of interest is that of Wireless Sensor Networks (WSN). WSN are networks extended over large geographical regions that feature low-power sensors which are interconnected to each other to receive and transmit data. In [23], an event-based sensor data scheduler deriving an approximate minimum mean-squared error (MMSE) estimator has been developed for remote state estimation over a network. In [24], the sensor selection problem over WSN was addressed using Kalman filters with various cost functions and network constraints and assuming a predetermined time horizon. Finally, a distributed sensor node-level energy management approach for minimizing energy usage was outlined in [25], which is based on target trajectory prediction through Kalman filters and Interacting Multiple Model (IMM) filters.

In contrast to all the above solutions, we propose a *black-box* approach to the selection of a subset of sensors, applicable to a generic indoor environment, and also well-suited for non-linear process models and time series consisting of both real and discrete values. Although we focus on the temperature estimation scenario, the approach can, in principle, be applied to any other prediction task.

## 3. The Considered Setting

**Figure 1:**
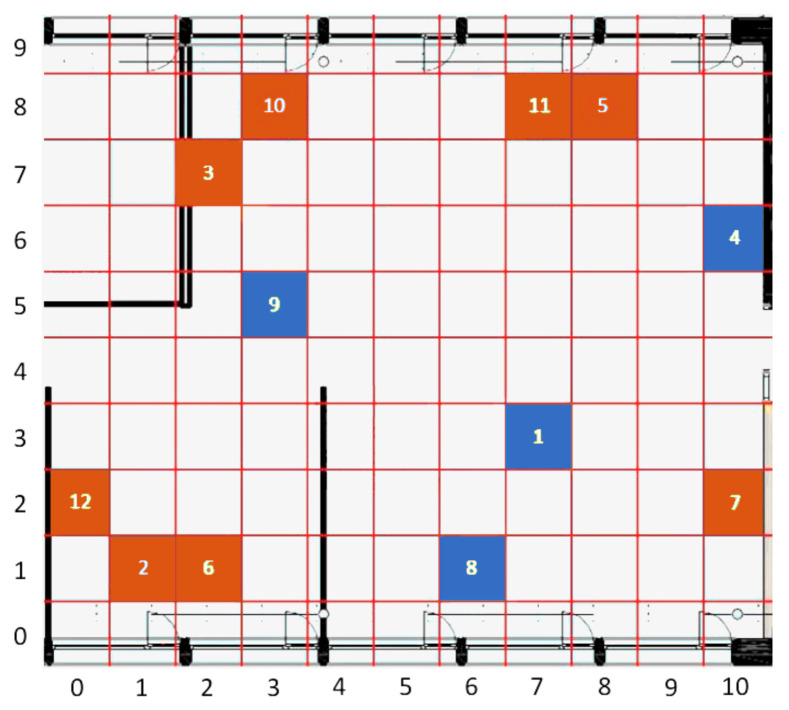
Location of the sensors in the considered premise. The blue cells represent the reference sensors that will be selected during the sensors selection phase.

**Figure 2:**
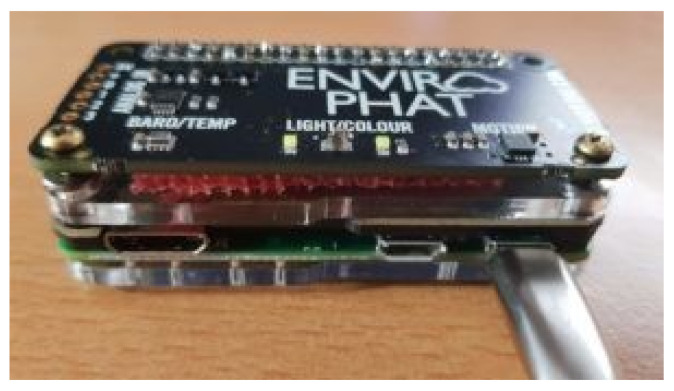
One of the Raspberry Pi Zero boards used in the work.

The considered scenario is an open-space office at Silicon Austria Labs, in Villach (Austria). As shown in Figure 1, the room is fairly large, having an overall surface of 127 m${}^{2}$, and it is characterized by the presence of an always-on air-conditioning system, intermittently used workplaces equipped with high-performance workstations, individually controlled radiators, and some windows, at the top and the bottom of the map, which can be independently opened and blinded. Twelve Raspberry Pi Zero boards (Figure 2) are deployed for the measurements, each one equipped with an Enviro pHAT sensor board featuring temperature, pressure, light, color, motion, and analog sensors. The recordings are transmitted via WLAN, through a FritzBox access point, towards a Raspberry Pi 3, which acts as a database server. The clients do not store any data: all measured values are sent immediately to the database with a preset periodicity of approximately 10 s. Both servers and clients run on Raspbian OS, while the server-side database is based on MySQL. Each client is programmed through a Python 3 script.

The placement of sensors has been organized with the goal of monitoring as large a variety of operating conditions as possible. As an example, sensor 9 is placed near the center of the room, and thus ought to be less affected by weather conditions than, for instance, sensors 2, 5, 6, 8, and 10, which are located close to a window. A grid has been superimposed over the map, which allows us to assign to each sensor a unique coordinate, expressed by a tuple (X,Y), with 0≤X≤10 and 0≤Y≤9.

The considered dataset consists of measurements taken from the twelve sensors over the period from 23 October 2019 to 3 March 2020. Data are recorded every 10 s, for a total of around 13,500,000 instances. The maximum observed temperature (Celsius) was 48.95, while the minimum was 5.36. This confirms the impact of weather: while, in the first case, the high temperature can be explained by both the sun influence and the temperature of the Raspberry circuitry, in the latter case it is likely to be caused by an open window placed in close proximity to one of the sensors.

## 4. Descriptive Analysis

**Figure 3:**
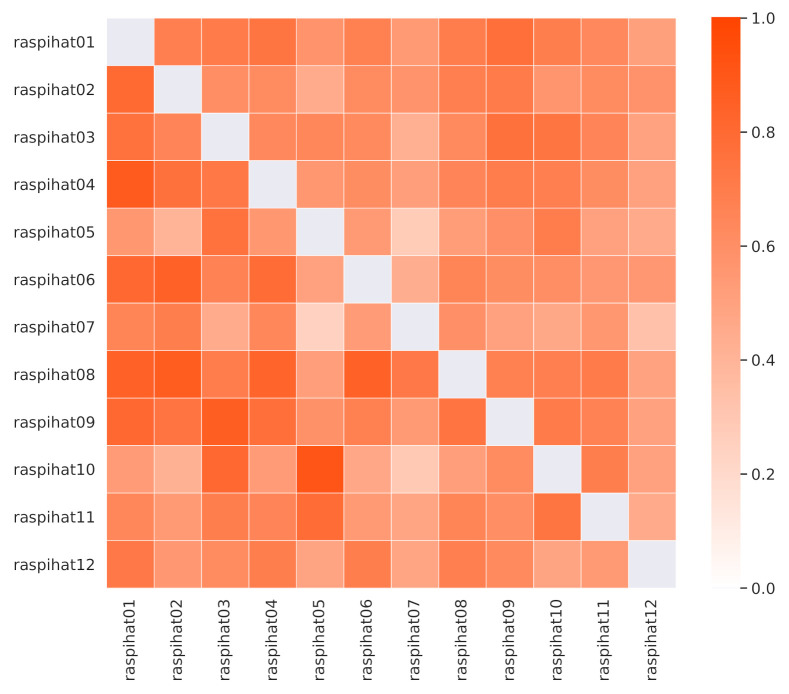
Pearson (lower triangular part) and Kendall (upper triangular part) correlation values among the recorded temperatures.

The Pearson correlation values among sensor temperatures are depicted in the lower triangular part of Figure 3, and they show that some of them are naturally more correlated than others. This is the case, for instance, with sensors located close to a window in the upper part of the room. Moreover, sensor 9 shows a high correlation with sensor 1, which is not surprising, as both of them are placed near the center of the room. However, there are also some notable exceptions. As an example, sensor 10 correlates more with sensor 5 than with sensor 3, despite the fact that it is much closer to the latter than to the former. A similar pattern can be observed for the pair of sensors 12 and 1, as opposed to 12 and 2. These phenomena might be explained by the proximity of a window, the heat emitted by desk lights or other kinds of electrical device, or by the presence of some obstacles that may interfere with air flow, or block the light reaching a specific sensor.

**Figure 4:**
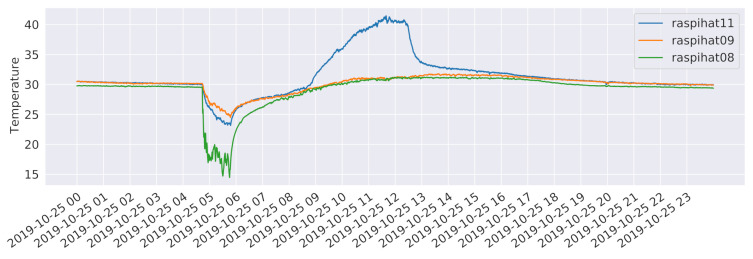
Temperatures recorded by sensors 8, 9, and 11 on the day 25 October 2019.

In order to investigate whether these pairs of neighboring sensors share some non-linear relationships or are simply measuring different phenomena, a further analysis based on Kendall’s tau, a non-linear rank correlation measure, has been carried out. Results are depicted in the upper triangular part of Figure 3. In this case, the correlation between the pair of sensors 12 and 2 is higher than the one estimated for the pair of sensors 12 and 1. Conversely, sensor 10 still correlates more with sensor 5 than with sensor 3, which, not being directly adjacent to a window, is probably measuring a different kind of phenomenon. As for the temporal evolution of the temperatures, Figure 4 shows the measurements of three different sensors for the day 25 October 2019, which follow a typical pattern along a 24 h period. It is worth noticing that the temperature recorded by sensor 11 has a spike around 11:30 a.m. in the morning. This is probably due to the heating effect of direct sunlight, that is not present on sensor 8, which is close to another window but on the opposite side of the building. Instead, the latter sensor shows a drop in the temperature at around 5:00 a.m., which can be explained by the daily cleaning staff operations, which include opening the nearby windows to circulate air. Finally, sensor 9 is characterized by a rather stable behaviour, being placed close to the center of the room. It is worth pointing out that, as witnessed by the high average temperatures, sensors have not been calibrated. This was done on purpose, in order to evaluate the performance of the proposed methods in a more challenging scenario, also considering that a proper calibration process might not always be possible in a real-world deployment.

As we shall see, in the remainder of the work we will always consider an 80–20% training-test split of the dataset, randomly assigning entire weeks to the two partitions. Such a choice has been made taking into account the fact that the recordings span less than one year and cover months that typically exhibit considerable meteorological variations. Thus, it would otherwise have been difficult for the machine learning approaches that we are going to consider to learn models over a subset of the first days, which are capable of generalizing well to the remaining time period. In addition, upon data inspection, it emerged that even contiguous weeks tend to have rather different behaviours.

The performed analysis allows us to conclude that the considered setting is not trivial, as there are some irregularities and local phenomena that influence and differentiate the temperature recordings of the different sensors, making the temperature prediction task quite challenging.

## 5. Data Pre-Processing

**Figure 5:**
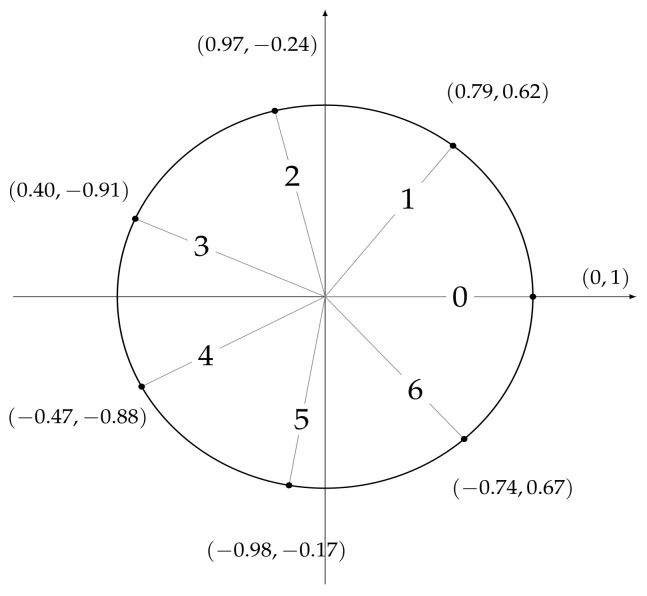
Values assigned to (*dow_sin*, *dow_cos*) after a trigonometric transformation of the *dow* feature, where *dow* ranges from 0 (Monday) to 6 (Sunday).

**Figure 6:**
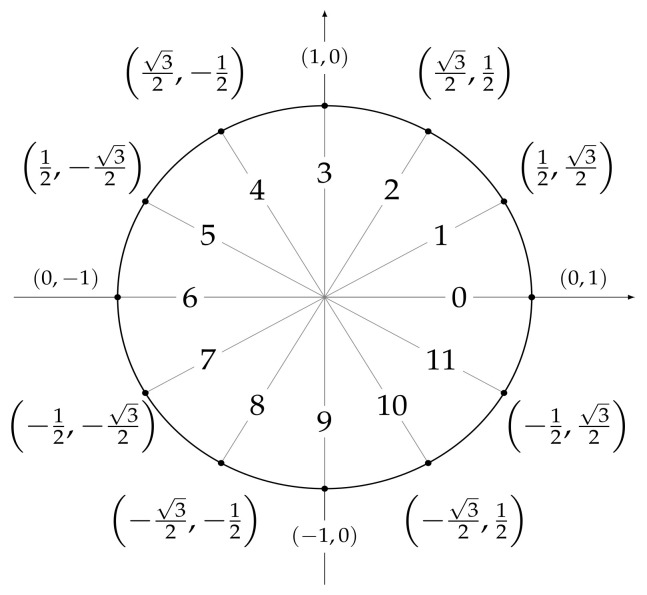
Values assigned to (*moy_sin*, *moy_cos*) after a trigonometric transformation of the *moy* feature, where *moy* ranges from 0 (January) to 11 (December).

**Figure 7:**
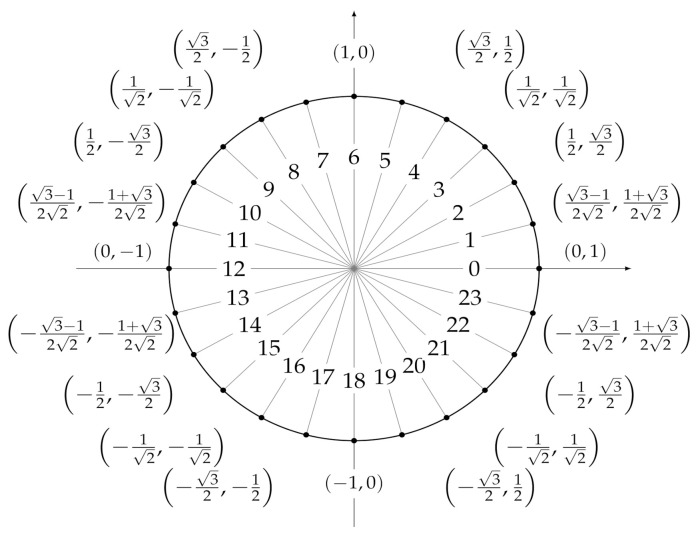
Values assigned to (*sec_from _midnight _sin*, *sec_from_mi-dnight _cos*) after a trigonometric transformation of the *sec_from_mid-night* feature ranging from 0 to 86,399.

In order to carry out the experiments, it is necessary to define a set of features that can be used as predictors by the machine and deep leaning models we are going to develop. To this end, for each temperature measurement, we considered a set of temporal attributes, that are useful to locate the observation in time. They are *sec_from_midnight*, which tracks the number of seconds elapsed from 00:00:00 h, *dow*, a numerical identifier of the day of the week, and *moy*, a numerical identifier of the month of the year. In order to account for time periodicity (and be able to consider, e.g., the fact that 11:59:59 p.m. is close to 00:00:00 a.m.), we encoded each feature by means of two trigonometric transformations

$$\begin{array}{c} \sin(2*\pi *x/\delta )\;\;and\;\;\cos(2*\pi *x/\delta ), \end{array}$$

where *x* represents the original attribute value and δ is the length of the period, e.g., 12 for the attribute *moy*. As a result, six features were obtained: dow_sin and dow_cos, whose values are shown in Figure 5, moy_sin and moy_cos, whose values are shown in Figure 6, and and sec_from_midnight_sin and sec_from_midnight_cos, whose values are shown in Figure 7.

### 5.1. Distance Metrics

The main goal of the work is to study a solution capable of exploiting a subset of sensors to make predictions in place of a sensor to be virtualized. To achieve this, distance metrics can be useful to decide which physical sensors to consider as predictors, and to compute the predicted temperature. Furthermore, distance metrics may be quite useful for the task of optimizing the positioning of a given set of sensors within a certain environment.

The following distance metrics between two sensors, $sensor_{i}$ and $sensor_{j}$, whose spatial positions are, respectively, $p_{i}:=\left(x_{i},y_{i}\right)$ and $p_{j}:=\left(x_{j},y_{j}\right)$, have been considered

- **Euclidean distance**: length of a line segment between the two points $p_{i},p_{j}$ defined as the $L_{2}$-norm $\sqrt{{\left(x_{i}-x_{j}\right)}^{2}+{\left(y_{i}-y_{j}\right)}^{2}}$;
- **Manhattan distance**: the $L_{1}$-norm of the distance, defined as $|\left(x_{i}-x_{j}\right)\left|+\right|\left(y_{i}-y_{j}\right)|$;
- **Chebyshev distance**: the $L_{\infty }$-norm of the distance, defined as $\max\{|\left(x_{i}-x_{j}\right)|,|\left(y_{i}-y_{j}\right)|\}$;
- **Genetic Programming distance**: a combination of the previous three distances obtained by means of a genetic programming algorithm which generates a computation tree whose leaves may contain the three aforementioned distance values or a randomly generated constant and whose internal nodes are the scalar/vector operations defined as a set of primitives;
- **Pearson correlation**: it expresses a possible linear relationship between the statistical variables given by the temperature values of the two sensors, and it is defined as
  $$\left(\sum_{k=1}^{n}\left(t_{i}^{k}-\bar{t_{i}}\right)\left(t_{j}^{k}-\bar{t_{j}}\right)\right)/\left(\sqrt{\sum_{k=1}^{n}{\left(t_{i}^{k}-\bar{t_{i}}\right)}^{2}}\sqrt{\sum_{k=1}^{n}{\left(t_{j}^{k}-\bar{t_{j}}\right)}^{2}}\right),$$
  where *n* is the sample size, $t_{i}^{k}$ and $t_{j}^{k}$ are the individual sample points for $sensor_{i}$ and $sensor_{j}$, that is, the temperature values, and $\bar{t_{i}}$ and $\bar{t_{j}}$ are the sample means;
- **Kendall correlation**: it expresses a possible ordinal (non-linear) association between the statistical variables given by the temperature values of the two sensors, and it is defined as
  $$\frac{2}{n(n-1)}\sum_{k<l}sgn\left(t_{i}^{k}-t_{i}^{l}\right)\;sgn\left(t_{j}^{k}-t_{j}^{l}\right)$$
  where *n* is the sample size, $t_{i}^{k}$ and $t_{i}^{l}$ are the individual sample points for $sensor_{i}$ in position *k* and *l*, $t_{j}^{k}$ and $t_{j}^{l}$ are the individual sample points for $sensor_{j}$ in position *k* and *l*, and sgn(x) is the function sgn:R→{−1,0,1} that returns −1, if x<0, 1, if x>0, and 0 otherwise;
- **SHAP distance**: SHAP is a game-theoretic method that allows one to evaluate the contributions to the final result of the different predictors used in a machine learning model, with the relevance of the contribution of a predictor to the model being proportional to its SHAP value [26]. In our case, SHAP values for a generic sensor $sensor_{i}$ are obtained from an XGBoostRegressor model [27] that predicts the temperature value of $sensor_{i}$ on the basis of the temperature values of the other sensors. It is worth noticing that such a metric is not symmetric;
- **SAX-CBD distance**: SAX-CBD is a Compression-Based Dissimilarity measure [28] based on the assumption that the size of the compressed file of the concatenation of two discrete time series is inversely proportional to the number of patterns that they share. As a preliminary step, the temperature values obtained from $sensor_{i}$ and $sensor_{j}$ are discretized by means of Symbolic Aggregate approXimation (SAX) [29]; then, the value of the distance is computed as
  $$size\_of\left(compress\left(d_{i}\;+\;\;\;+\;d_{j}\right)\right)/\left(size\_of\left(compress\left(d_{i}\right)\right)+size\_of\left(compress\left(d_{j}\right)\right)\right),$$
  where ++ is the concatenation operator, $d_{i}$ and $d_{j}$ are the time series consisting of, respectively, the discrete temperature values related to $sensor_{i}$ and $sensor_{j}$, and compress (data) are the output of the application of the algorithm DEFLATE [30] to data.

The estimation of the above metrics is straightforward and it has been made considering only the training set data, in order to obtain an ordering of the remaining 11 sensors, from the closest to the farthest one. These ranks are calculated for each distance metric listed above.

The genetic programming algorithm was designed relying on the Distributed Evolutionary Algorithms in Python (DEAP) framework [31], considering 100 generations with a population of 600 individuals, i.e., computation-tree-encoded functions. As for the evolutionary operators, we employed one point crossover, with a probability equal to 0.7, and a mutation, with probability 0.4, where a randomly chosen primitive from an individual is replaced by another randomly chosen operation within the primitive set. The chosen selection method is the double tournament [32], which evaluates both the fitness and the size of the individuals in order to discriminate good solutions, following a three-individuals fitness-based first tournament and a size-based second tournament with a parsimony size of 1.4. This last tournament favours the choice of low-complexity solutions, represented by trees of limited height. Individuals are built considering the three (0–1 normalized) distance metrics *Euclidean distance, Manhattan distance*, and *Chebyshev distance*, and a set of random constants ranging from −1 to 1 as terminal leaves. The set of primitives consists of the following scalar/vector operations: $\min,\max,+,-,*,÷,\log_{10}$, *exponentiation*, *square_root*, *negation*, and *absolute_value*. Note that, despite the normalization step performed on the distance metrics, the *absolute_value* operation is still useful given the presence of potentially negative constants in the tree.

**Figure 8:**
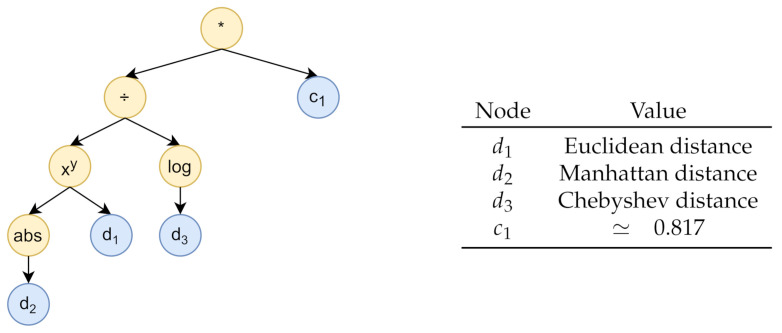
The computation tree generated by the genetic programming algorithm.

In order to determine the fitness function, we considered a further 90–10% training-validation subsplit of the training data, randomly assigning entire weeks to the two sets. In both datasets, each instance consists of a label, that is, the temperature recording of a given sensor, and a list of predictors, which are the 11 temperature values of the other sensors, and the temporal features *sec_from_midnight_sin*, *sec_from_midnight_cos*, *dow_sin*, *dow_cos*, *moy_sin*, and *moy_cos*. The fitness function was computed for each individual as follows: we assessed the prediction error obtained from a series of linear regression models built on the training split and evaluated on the validation split; different models were trained and evaluated, considering each different sensor as a target and increasingly discarding other predictor sensors according to the rank defined by the distance function encoded by the individual, starting from the sensor with the highest value. Then, for each number of considered predictors, we summed the resulting prediction errors, coming from the different target sensors, obtaining an error curve. Finally, to determine the fitness value, we calculated the area under the curve. The computation tree generated by the genetic programming algorithm is shown in Figure 8. It is equivalent to the function $GP\_function\left(d_{1},d_{2},d_{3}\right)=\left(\right|d_{2}{|}^{d_{1}}/log_{10}\left(d_{3}\right))*c_{1}$, where $d_{1}$ is the *Euclidean distance*, $d_{2}$ is the *Manhattan distance*, $d_{3}$ is the *Chebyshev distance*, and $c_{1}≃0.817$.

The evaluation of the rankings generated by each distance metric *d* was carried out according to the following procedure on the 80–20% training-test split:

- For each sensor $sensor_{i}$, the rank $rank_{d,i}$ of the other sensors according to the metric *d* was considered;
- then, we proceeded in an iterative way: for k∈{0,⋯,10}, *k* sensors among the worst ones in $rank_{d,i}$ were discarded and a regression model was built. In more detail,
  - The sensors whose temperature values were to be used as predictors were determined considering the set of all sensors, except $sensor_{i}$ and the *k* sensors located in the last *k* positions of $rank_{d,i}$;
  - Exploiting the training set data, a linear regression model was built to predict the temperature of $sensor_{i}$ using as input the temperature values of the sensors selected in the previous step and the features *moy_sin, moy_cos, dow_sin, dow_cos, seconds_from_midnight_sin*, and *seconds_from_midnight_cos*;
  - The resulting model was evaluated on the test set.

For each value of *k*, the sum of the 95th percentile of the absolute errors obtained for each predicted sensor was computed, obtaining a curve over *k*. Then, the final error for each metric was determined by calculating the area under the considered curve.

**Figure 9:**
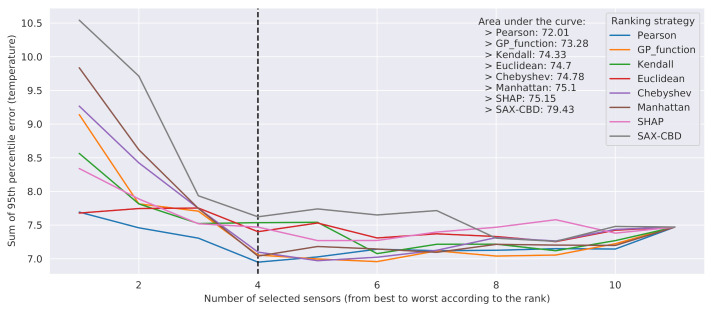
Performance of linear regression, evaluated discarding sensors based on training set ranks. The dashed vertical line represents the elbow of the Pearson error graph.

The outcome of the evaluation is reported in Figure 9. The curves show a bowl-shaped pattern. This can be explained by the fact that the first discarded sensors may have little correlation with the temperatures to predict, and thus they may interfere with the accuracy of the final result. On the other hand, the lastly discarded ones were probably carrying useful information. The best metrics turned out to be *genetic programming distance* and *Pearson correlation*.

More precisely, *genetic programming distance* provided the best results when considering a subset of at least five sensors as predictors and, as expected, generally outperformed all the other metrics based exclusively on spatial distances. The *Pearson correlation* showed an overall better performance than *genetic programming distance*, in particular when four sensors or fewer were considered as predictors. However, *Pearson correlation* cannot be employed in a more general setting, that is, to determine the best sensors for use as features to predict the temperature at a given cell, where a reference physical sensor may or may not be present. As for the *genetic programming distance*, while we cannot exclude its ability to generalize on each cell, the latter should be actually demonstrated, and it will be the subject of future investigations based on physical data simulations. As a last remark, we observe that Figure 9 clearly shows that excellent results, in terms of prediction error, can be obtained from just a subset of 4–6 sensors, while they become worse when considering three sensors or fewer.

### 5.2. Sensors Selection

In order to automatically select a subset of sensors to be used as predictors, we need to specify a procedure to determine the number $n_{refs}$ of sensors to select and to establish which sensors to actually consider. To this end, we make use of a procedure based on the Borda count voting method [33]. As a preliminary step, let us introduce some auxiliary notions. First, for i∈{1,⋯,12}, let $rank_{i}$ be the ranking of sensor *i* obtained by sorting in descending order the remaining 11 sensors according to their Pearson correlation with reference to sensor *i*. Then, let us define $w_{i}$, with i∈{1,⋯,12}, as the weight of $rank_{i}$, defined as $1-(\varepsilon_{i}/\max_{i\in \{1,\cdots ,12\}}\varepsilon_{i})$, where $\varepsilon_{i}$ is given by the sum of the 95th percentile of the absolute errors evaluated for $rank_{i}$, computed training different LinearRegression models, varying the number of sensors used as predictors from 1 to 11, as described for the case of the metric evaluation procedure at the end of the previous section (Section 5.1).

**Figure 10:**
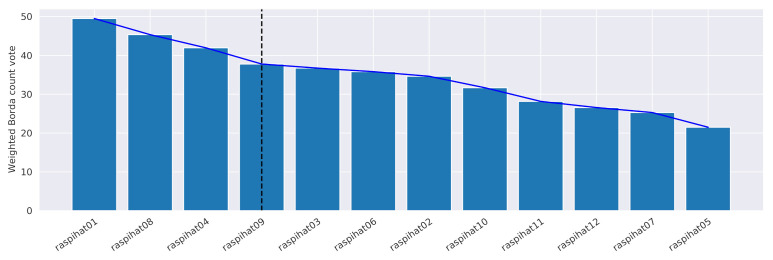
Weighted Borda count vote for each sensor. The vertical line represents the elbow of the graph, and it separates the selected sensors from the discarded ones.

The procedure consists of the following five steps: (*i*) for each sensor *i*, we compute $rank_{i}$; (ii) for each sensor *j*, we determine its weighted Borda count, which is defined as $vote_{j}=\sum_{i\in \{1,\cdots ,12\},\;i\neq j}\left(n_{sensors}-pos_{i}\left(j\right)\right)\cdot w_{i}$, where $pos_{i}\left(j\right)$ is the position of sensor *j* in $rank_{i}$, $n_{sensors}=12$ is the total number of used sensors, and $w_{i}$ is the weight of $rank_{i}$; (iii) the sensors are sorted in descending order according to their final weighted Borda count vote; (iv) an approximation of the elbow of the curve obtained in the previous step is computed by using the Kneedle algorithm [34]—the *x*-axis value corresponding to the elbow represents the point of maximum curvature of the graph, and the best trade-off between prediction accuracy and number of sensors, after which a law of diminishing returns applies: we choose it to be the $n_{refs}$ value which, in our case, corresponds to four reference sensors (dashed line in Figure 10); (*v*) finally, the first $n_{refs}$ sensors are selected as the reference ones. It is worth underlining that the weights $w_{i}$ in the vote-counting formula allow us to offer greater importance to the ranks that provided better results with respect to the error obtained on the validation data split. In addition, the sensors located on the left side of elbow intuitively correspond, by construction, to the best predictors.

Figure 10 shows the weighted Borda count votes for all sensors obtained by applying the procedure to the considered setting. The proposed criterion led to the selection of sensors 1,8,4, and 9 as the references for the prediction. It is noteworthy that the elbow estimate also corresponds to the minimum error point of *Pearson distance* in Figure 9. Even if, in our case, no ex aequo placements occur, as a methodology to deal with them, we suggest to consider, for each sensor, the median of its Pearson correlations with respect to the other ones, prioritizing those with higher values.

### 5.3. Feature Selection

On the basis of the feature engineering and sensor selection phases, we identified 16 attributes that describe each observation: the temporal features *sec_from_midnight_sin*, *sec_from_midnight_cos*, *dow_sin*, *dow_cos*, *moy_sin*, *moy_cos*, the spatial features *01_ref_dist*, *04_ref_dist*, *08_ref_dist*, *09_ref_dist*, and the reference temperatures *01_ref_temp*, *04_ref_temp*, *08_ref_temp*, *09_ref_temp*. To them, we added *X_coord* and *Y_coord*, that is, the two grid coordinates of the sensor that recorded the observation.

**Figure 11:**
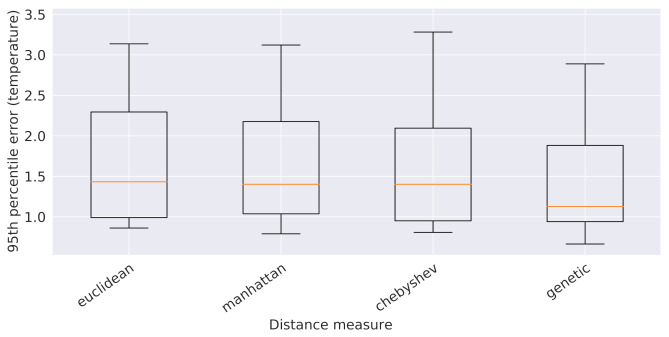
Boxplots of the 95th percentile of the error provided by the XGBoost models built on the 5 spatial distances considered in this work.

At this point, it is necessary to establish which spatial distance metric to consider among *Euclidean distance*, *Manhattan distance*, *Chebyshev distance*, and their combination, obtained via *genetic programming*, for the estimate of *01_ref_dist*, *04_ref_dist*, *08_ref_dist*, and *09_ref_dist*. To this end, we determined the 95th percentile of the validation error values obtained from 5 global 16-attribute XGBoostRegressor models, one for each spatial distance metric, trained on the usual 90% subsplit of the original training data pertaining to all the sensors, except for the reference ones (which are already used as predictors). The outcome of such an analysis is depicted in Figure 11 and led to the selection of the *genetic programming distance*, which outperformed all the other ones. At the end, the following 16 attributes were chosen to describe each observation: the 6 temporal features, the 4 reference temperatures, the 4 spatial features *01_ref_gpdist*, *04_ref_gpdist*, *08_ref_gpdist*, and *09_ref_gpdist*, which are the genetic programming distances from the reference sensors, and the 2 grid coordinates *X_coord* and *Y_coord* of the sensor that recorded the observation.

**Figure 12:**
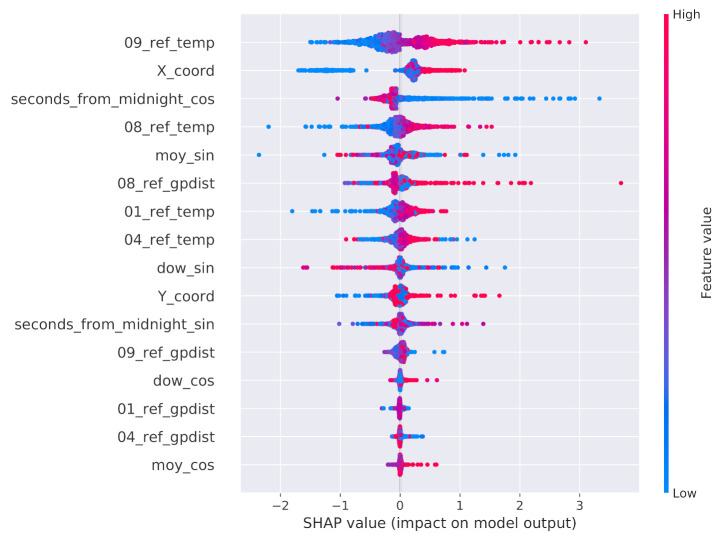
SHAP values of the attributes considered in the second step of the feature selection process.

Since some of these attributes may be redundant, we executed a two-step feature selection process working on the training split. As a preliminary data preparation step, all attributes were standardized by subtracting their mean and dividing by their standard deviation. The first selection step searched for highly correlated attributes, that is, attributes with a Pearson correlation value above 99%. As a matter of fact, no feature was removed from the dataset by this step. The second step evaluated the potential impact of the remaining attributes on the final prediction. To this end, the SHAP values extracted from a single global XGBoostRegressor model trained on the training data split related to all the sensors, except for the reference ones, were taken into consideration. The outcome of such an analysis is depicted in Figure 12. We first observe that all reference temperature values have a large impact on the final prediction and, naturally, higher values of these attributes increase the value of the prediction. Focusing on the office map, there is a clear variation across the *X* and *Y* axes. As for the *Y* axis, from the distribution of the values, we can conclude that, during the data collection period, the northern side of the room was generally warmer than the southern one. As for the *X* axis, the eastern side of the room seems to be warmer than the western one. Interestingly, the moy_cos and dow_cos features do not seem to be important for the overall prediction when compared to the counterpart obtained from the sine transformation. As pointed out by Figure 5 and Figure 6, this means that the contribution to the prediction given by the features that discriminate the first half of the week/year from the second half are more important than those that discriminate the first/fourth quarters from the second/third ones. Furthermore, the genetic programming distances from sensors 1, 4, and 9 are considered of marginal importance when compared to the distance from sensor 8. On the basis of the SHAP results, we ultimately decided to remove the five attributes *dow_cos*, *moy_cos*, *01_ref_gpdist*, *04_ref_gpdist*, and *09_ref_gpdist*, ending up with a total of eleven attributes.

From a general point of view, the first correlation-based feature selection step must be considered as a preliminary, coarse screening of the predictor variables; it should discard a feature when it is found to be almost identical to another one in the dataset, without the risk of removing predictors that might still be preserved by the subsequent, SHAP-based feature selection step. This is the reason that we recommend relying on a high threshold, which should nevertheless be established considering the specific scenario. Indeed, such a pre-screening may be useful in a more general situation, characterized by a large amount of input features, to reduce the time requirements needed to train the XGBoost model in the second step of the feature-selection phase. In our case, as already discussed, relying on a correlation threshold of 99% led to no attribute being discarded. However, lowering the threshold to 94% would have led to the removal of feature *04_ref_gpdist*, which would also have been discarded in the subsequent SHAP-based step. To remove the first feature kept in the SHAP-based step, namely *04_ref_temp*, the correlation threshold should have been reduced to a value less than or equal to 88%.

## 6. Predictive Analysis

To determine the performance of the sensor and feature selection phases with respect to the task of temperature virtual sensing, and to identify the best prediction methodology, we experiment with and contrast the following approaches:

- Baseline: simple and Inverse Distance Weighted (IDW) average of the temperatures;
- Particle filters;
- LinearRegression – Python’s package Scikit-learn [35];
- XGBoostRegressor – Python’s package xgboost [27];
- An LSTM recurrent neural network, trained by means of the PyTorch Deep Learning library [36].

As pointed out in Section 5.2, except for the baseline methods and particle filters, predictions make use of the temperature values recorded by the four chosen reference sensors (sensors 1, 4, 8, and 9) depicted in blue in Figure 1. To ensure the comparability of the results obtained from the various approaches, prediction errors were evaluated on the remaining eight sensors (the original 12, except the four reference sensors that are already used as predictors). In addition, as previously mentioned, we always considered the same 80–20% split training-test of the dataset.

The predictive analysis tasks are organized as follows. In Section 6.1, Section 6.2, Section 6.3 and Section 6.4 we evaluate the various approaches to temperature virtual sensing. Then, we analyze the prediction errors: Section 6.5 assesses the uncertainty associated with the predicted quantities, Section 6.6 discusses the errors per single sensor, and Section 6.7 links the prediction error to the available training data. Finally, in Section 6.8 the outlined framework is evaluated with respect to the optimal result that can be achieved by means of a brute force approach to sensor selection.

### 6.1. Baseline Methods

This first analysis allowed us to define a baseline against which to compare the results of the other approaches. Given a sensor for which we want to predict the temperature readings on the test set, the idea is that of approximating such values by a simple combination of the temperatures recorded by the other ones at the same time instant. To this end, we applied two different techniques: classical average and Inverse Distance Weighted Average (IDWA), according to which closer sensors have an impact on the overall prediction greater than that of sensors which are farther away. In more detail, the weight assigned to the *i*-th predictor is computed as $w_{i}=\frac{1}{d(x,x_{i})},$ where d(·,·) is the genetic-programming-based distance (Section 5.1) between two points of the grid, $x_{i}$ is the position of the *i*-th sensor, and *x* is the position of the sensor to predict. The weighted temperatures are summed and then divided by the sum of the weights.

**Figure 13:**
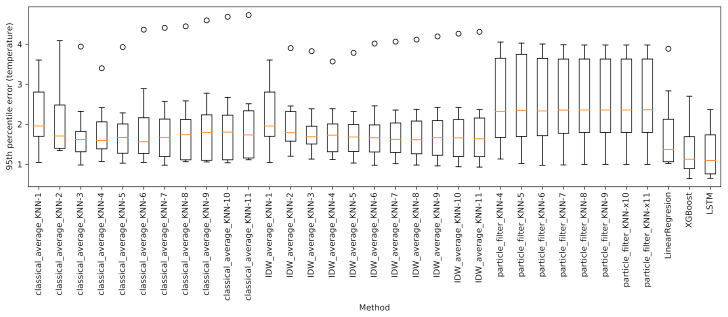
Boxplots of the 95th percentile of the error provided by the considered approaches.

Moreover, to determine the impact on the prediction accuracy of the distance between sensors, we performed several experiments by considering as predictors just the *k* sensors closest to the one to predict, for k∈[1,11]. For each approach and sensor to evaluate, we determined the temperature absolute error, considering its 95th percentile, $\epsilon_{95}$, which can be thought of as a worst-case prediction scenario. Figure 13 collects the boxplots of $\epsilon_{95}$. Each boxplot includes a value for every test tensor, for a total of eight values. The orange line represents the median of $\epsilon_{95}$, while the whiskers correspond respectively to the minimum and maximum values excluding the outliers (< 1st quartile − 1.5 IQR or > 3rd quartile + 1.5 IQR, where IQR is the interquartile range given by the difference between the 3rd and the 1st quartile). It clearly emerges that classical average is largely influenced by the number of closest sensors used for the prediction. Here, the optimal number of sensors seems to lie in the range [3,5]. On the other hand, IDWA seems to be less affected by the number of predictor sensors. Indeed, looking at the median, large values of *k* led to better results. This is to be expected, as the contributions of the different sensors are already weighted according to their distances.

### 6.2. Particle Filters

Particle filters are a class of Sequential Monte Carlo algorithms used to approximate the internal states of dynamic systems starting from partial measurements with random disturbances, which afflict the sensors as well as the dynamic system itself [5]. Given the noisy and partial observations, this approach aims at measuring the state posterior distributions of some Markov process. Particle filters leverage a set of particles to represent such a posterior distribution. Each particle has an assigned weight, indicating the chance of that particle being sampled from the probability density function of the quantity we want to compute. As for this experimentation, we considered one particle filter for each of the eight evaluated sensors, and the *k*-closest sensors as landmarks, with k∈[1,11]. Each particle thus represents a likelihood estimation of a temperature and is moved at each time-step following the average temperatures of the landmarks. The likelihood probability is computed on the basis of the genetic programming distance between particles and landmarks.

As with the baseline approaches, we estimated the error’s 95th percentile for each evaluation sensor and each potential *k* value. The results are reported in Figure 13. It is evident that particle filters are also affected by the choice of *k*. For the sake of readability, we decided to ignore the results for k∈[1,3], as those values led to very weak predictions, with boxplot whiskers extending over six degrees. As for the remaining values, according to the median, the best predictions are achieved for values of *k* equal to 4 or 5.

### 6.3. Machine Learning Approaches

We considered two different machine learning approaches, namely, a simple Scikit-learn’s LinearRegression model, and a more complex XGBoostRegressor ensemble approach.

LinearRegression implements an ordinary least squares linear regression. For the sake of our study, it has been trained on the 11 (standardized) features selected in Section 5, with training labels corresponding to the temperatures recorded by eight evaluation sensors (the original 12, except the four reference sensors that are used as predictors).

XGBoost [27] implements gradient-boosted decision trees focusing on computational speed and model performance. Gradient boosting iteratively builds new models to predict the residuals of errors of previous models exploiting a gradient descent algorithm to minimize the loss [37]. The resulting models are then combined to generate the final prediction. As a first step, we tuned the XGBoostRegressor model with the above-described training set, from which a validation set of size 20%, consisting of randomly chosen weeks, was extracted. The task was performed by means of *Hyperopt* [38], a library for hyperparameter optimization written in Python, minimizing the 95th percentile of the error loss function for 40 evaluation steps on the following hyperparameters: max_depth, learning_rate, n_estimators, reg_alpha, reg_lambda, gamma, subsample, colsample_bytree, and min_child_weight. The resulting values are listed in Table 1. With the tuned hyperparameters, the model was trained on the entire training set, and then evaluated on the test set over the usual eight sensors.

The outcomes shown in Figure 13 suggest that the tested machine learning methods vastly outperform the baseline approaches. Specifically, XGBoost shows a better performance than LinearRegression on all 95th error quantiles. Furthermore, XGBoost’s boxplot is wider than that of LinearRegression, suggesting a more unstable behaviour across the sensors predictions. This is to be expected, being XGBoost a far more complex and flexible model.

### 6.4. Deep Learning Approach

**Figure 14:**
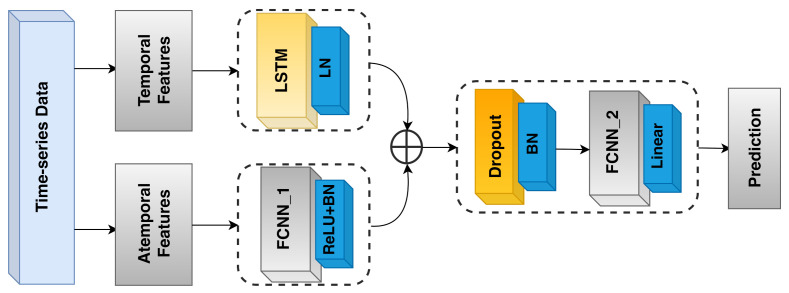
The LSTM recurrent neural network model architecture.

Up to this point, to predict the temperature at a given time instant, we considered reference sensor values from the same instant. In the literature on remote and virtual sensing, it has already been shown that deep learning methods are capable of taking temporal and spatio-temporal knowledge into account (see, e.g., [11,12,13]). Specifically, in our context, it may be the case that the recent history of temperatures reported by the reference sensors provides information that is relevant to the overall prediction. As an example, when opening a window in winter time, one may notice a regular and continuous decrease in the temperatures recorded by a nearby temperature sensor. This, together with information recorded by the other reference sensors, may give the model a hint regarding the temperature propagation in the room. We designed an LSTM-based model that takes such histories into account. Its architecture, which is depicted in Figure 14, consists of three subparts:

- The first (*temporal*) part (LSTM on the upper left side of Figure 14) takes a history of the four (standardized) reference temperatures as input. Then, a unidirectional LSTM layer, consisting of 128 units, from which we retrieve just the last outputs, followed by LayerNormalization, is applied;
- The second (*atemporal*) part (FCNN_1 on the bottom left side of Figure 14) takes the seven remaining (standardized) attributes as input, resulting from the feature selection process (Section 5). These attributes do not have any significant history, but are still important to generate the final output, since they allow the model to pinpoint the prediction in space and time. The aforementioned seven features are passed to a Dense layer, consisting of 64 neurons, followed by a ReLU activation function and a BatchNormalization layer;
- The third part (FCNN_2 on the right side of Figure 14) takes the outputs of the first two parts and concatenates them, generating a tensor of size 192. Then, BatchNormalization and Dropout with 0.1 rate are applied to the result of such a concatenation. Next, the data go through a dense layer of 128 units, followed by a ReLU activation function, and a BatchNormalization layer. The final output is produced by a single-unit Dense layer with linear activation function.

In order to train the neural network, we relied on Adam optimizer with a $9\times 10^{-5}$ learning rate minimizing the mean_squared_error loss function. The architecture of the model and all the other hyperparameters were chosen through iterative random search tuning performed on a fixed 80–20% training-validation subsplit, as was already done for the previous machine learning approaches. At last, the same tuning process suggested a length of 18 samples (equivalent to a period of 3 min) for the reference temperature histories, over a tested range of from 1 to 5 min.

As shown in Figure 13, with respect to the test set, the network essentially provided the same performance as XGBoostRegressor: while the upper whisker is marginally better than that of the ensemble approach, the lower one is slightly worse. Furthermore, the broader extension of the boxplot suggests a more variable behaviour than XGBoost. Based on these results, perhaps surprisingly, we can infer that, in our setting, historical knowledge of temperatures alone does not contribute much to the accuracy of the prediction. This can be justified by the fact that the sensors we want to predict are typically located much closer to a window than the reference ones, and thus they should also be the first to be affected by any weather-related phenomenon. Accordingly, reference sensors’ historical data are not so important from this point of view. As an additional confirmation, results of an auto-correlation analysis showed that information conveyed by the reference sensors at the time instant in which the prediction is carried out is way more relevant than information present in the historical temperature values related to the same sensors.

In future work, we plan to evaluate the performance of a CNN-LSTM-based neural network that, in principle, should allow us to relate the temporal dimension of temperature histories to spatial information about the placement of the reference sensors, the distances among them, and their distance from the location we want to predict.

### 6.5. Prediction Intervals Analysis

Usually, a regression model generates a single value for each prediction, which represents itself a random variable. However, under several circumstances, quantifying the uncertainty associated with the prediction, instead of computing just a single value, is very useful, as it gives an indication of the reliability of the results. This can be done by setting proper prediction intervals. Such intervals provide probabilistic upper and lower bounds on the estimate of an outcome variable and can be computed via quantile regression [39]. Typically, regressions minimize the mean squared-error loss function $L\left(y,\hat{y}\right)=\frac{1}{N}\sum_{i=1}^{N}{\left(y_{i}-\hat{y_{i}}\right)}^{2}$, while quantile regression aims at estimating conditional quantiles of the response variable. This is achieved by adopting the loss function $L_{\gamma }\left(y,\hat{y}\right)=\sum_{i=y_{i}<\hat{y_{i}}}^{N}\left(\gamma -1\right)|y_{i}-\hat{y_{i}}|+\sum_{i=y_{i}\geq \hat{y_{i}}}^{N}\left(\gamma \right)\left|y_{i}-\hat{y_{i}}\right|$, where *N* is the number of the samples in the training set, γ is the quantile of the response variable to forecast, $\hat{y_{i}}$ is the predicted value for the i-th sample, and $y_{i}$ is the real target value for the i-th sample.

**Figure 15:**
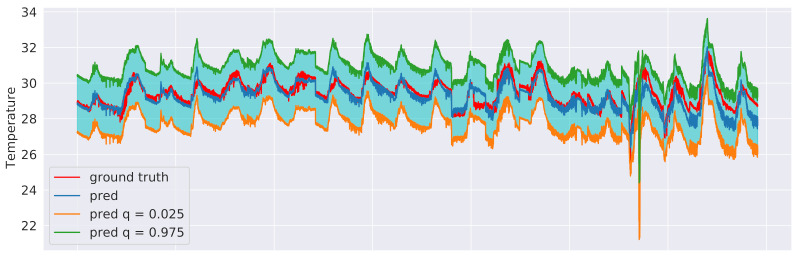
Linear regression prediction intervals related to sensor 3 test data with γ=0.025 and γ=0.975.

**Figure 16:**
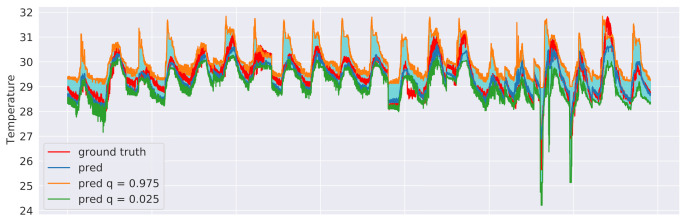
Gradient boosting regression prediction intervals related to sensor 3 test data with γ=0.025 and γ=0.975.

We explored two different approaches to quantile regression: a linear regression model and a gradient-boosting regression model [37]. In both cases, two models were trained: one for the upper (γ=0.025) and one for the lower (γ=0.975) bound of the interval. This means that 95% of the actual values should lie between these two predicted bounds. The training procedure was the same as in Section 6.3 and an excerpt of the results for the sensor 3 test data is shown in Figure 15, in the case of linear regression, and in Figure 16, in the case of gradient-boosting regression. Although both approaches were indeed observed to guarantee that 95% of the observed values end up between their estimated lower and upper bounds, the intervals obtained from the Gradient Boosting models are generally less coarse, thus providing a better approximation of the uncertainty intervals.

### 6.6. Errors per Single Sensor

**Figure 17:**
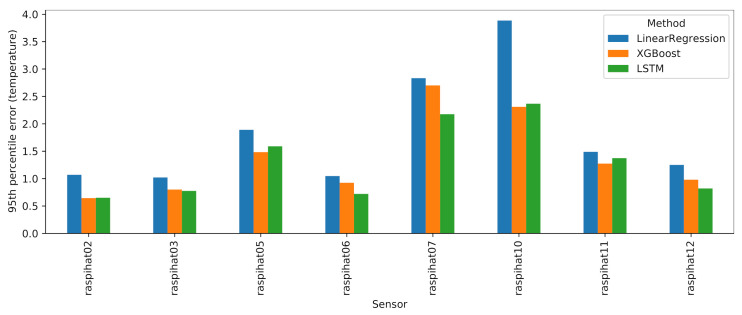
Results of machine and deep learning approaches for each evaluation sensor.

**Figure 18:**
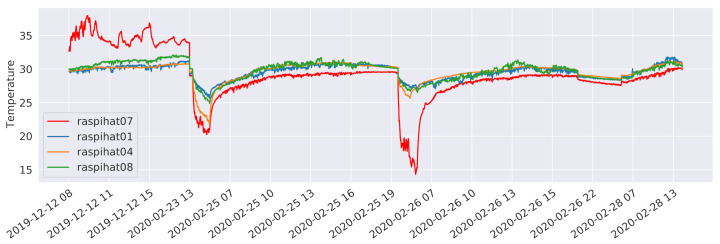
Sensor *raspihat07* temperature values related to the prediction error outliers compared with the three nearest-neighbour sensors.

Let us now take a closer look at the performance of the machine and deep learning approaches. Figure 17 reports the 95th percentile errors of such models for each of the eight evaluation sensors. Although the LSTM and XGBoost models typically exhibit a better performance than LinearRegression, the latter, despite being a much simpler model, is a close match, with the notable exception of sensor 10, where it is vastly outperformed by its contenders. It is also worth observing the relatively high error rates on sensor 7: upon closer inspection, this sensor displayed a very strange, fluctuating curve, as shown in Figure 18. It may be difficult to predict this kind of irregular temperature values using a global model for all eight sensors, as is done in this work. Therefore, as a future analysis, we plan to compare the outcomes of the global models with those that can be obtained by building a single model for each sensor to be predicted.

### 6.7. Effects of Reducing the Training Data Size

**Figure 19:**
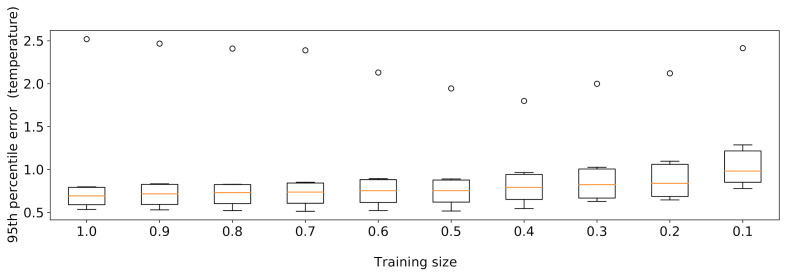
XGBoost results obtained by varying the training set size.

Given the large quantity of training data available, we examined the effects of reducing its size, relying on the XGBoost model introduced in Section 6.3. We tested various training set cardinalities obtained by sampling the data at one week granularity. The experiment was conducted using 10 sampling rates of r∈{0.1,0.2,⋯,1.0}, repeating each execution with 10 different random seeds to prevent sampling bias. As expected, the findings in Figure 19 show that, as the training data size decreases, the median of the 95th percentile of the errors raises, probably due to the fact that some seasonal trends may be overlooked if a too-small data sample is employed.

To further investigate the prediction error related to the outliers, we iteratively discarded the test data belonging to different devices, and it ultimately emerged that the anomalous values belonged to the predictions made for sensor *raspihat07*. Figure 18 shows the temperature values of sensor *raspihat07* and of its three closest neighbors (*raspihat01, raspihat04*, and *raspihat08*) limited to the time instants at which outliers are present in the prediction error (*mean absolute error* ≥2 ${}^{{}^{\circ}}$C). At these time instants, sensor *raspihat07* shows a more marked fluctuation behaviour for both high and low values, which may suggest a degradation or bad calibration of the device. Finally, we reran the training data size reduction experiment, discarding the data related to sensor 7 from the evaluation. In this way, a more predictable monotonic decrease in the prediction performance was observed.

### 6.8. Comparison with a Brute Force Approach

**Figure 20:**
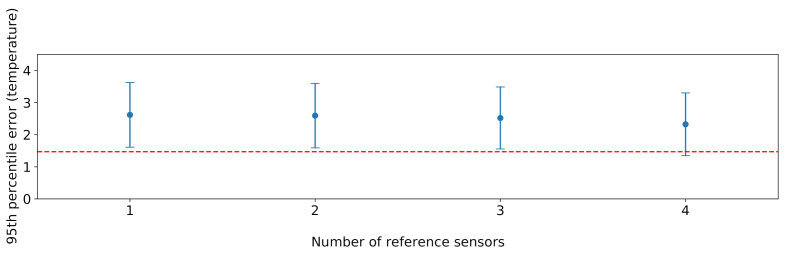
Results obtained from XGBoost on all possible combinations of *k* reference sensors, with k∈{1,⋯,4}. For each value of *k*, the vertical line represents the extent of the errors given by the different combinations, while the dots represent the median error. The red dashed horizontal line represents the error obtained by the subset of reference sensors selected by our approach.

Last but not least, in order to evaluate the effectiveness of the proposed sensor selection procedure, we carried out a comparison with a brute force approach which considers any possible combination of four sensors or fewer, chosen as the reference ones. For each possible combination, we trained an XGBoost model on the training data split, based on the features discussed in Section 5.3, before the feature selection phase. To ensure comparability, we performed our evaluation considering the test data split of the four sensors 2, 5, 7, and 10, since they cover the whole room and are also typically placed in the lower tier of the rankings analysed in Section 5.1. Excluding the aforementioned four sensors, the possible reference sensor combinations are ∑k∈{1,⋯,4}8k. As for the cardinality *k* of the subset of selected reference sensors, a maximum value of four was chosen to ensure that there is no combination with a lower number of sensors capable of providing a better prediction accuracy than the one obtained with the proposed solution. Results are shown in Figure 20. The performance of the proposed solution is very close to the optimal one achieved by brute force. This is remarkable, especially considering that the brute force solution performs an exhaustive search in the problem space, and can thus only be applied in scenarios involving a small number of sensors.

## 7. Discussion

In the previous sections, we focused on how to perform virtual sensing efficiently, dealing with the problem from different perspectives. While it is clear that prediction performances are expected to improve, together with the number of sensors and historical training data available, it is typically worth finding a trade-off between the accuracy of the models and the cost of the employed resources.

Our proposed solution encompasses all the relevant aspects of virtual sensing, including sensor selection, the estimatation of the needed amount of training data, the choice of predictive model, and the evaluation of its performance. Most importantly, the approach can be regarded as a *black-box*, completely independent from the physical characteristics of the considered scenario, such as any element capable of influencing the internal temperature (windows, radiators, etc.). Thus, in principle, it can be applied to any generic indoor environment with an arbitrary set of pre-located sensors.

**Figure 21:**
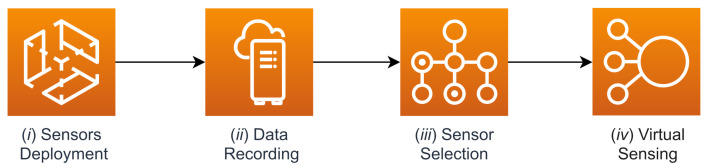
High-level steps which characterize the proposed procedure.

Here, we provide a final overview of the overall procedure, whose steps, as portrayed in Figure 21, are as follows: (*i*) a set of sensors is placed inside a room; (*ii*) the temperature values measured by these sensors are collected over a significant period of time; (*iii*) following the steps outlined in Section 5.2, a subset of sensors is selected from those present in the room by exploiting the Pearson correlation of their temperature measurements; (*iv*) the other sensors can be removed from the room and replaced by the output of predictive models built using information from the remaining reference sensors, as shown in Section 6.

Specifically, it is once again worth highlighting the role played by the most important component of the procedure, i.e, the proposed sensor selection technique, which provided a very good result when compared to a plain brute force approach. This could be achieved leveraging the weighted Borda count method in combination with the Kneedle algorithm: the former allows us to give a greater importance to the sensor ranks that provided the better results, thus favoring sensors that ought to be the most relevant predictors; the latter reasonably approximates a trade-off point at which the cost of increasing the number of sensors used as predictors is no longer worth the corresponding performance benefit.

## 8. Conclusions and Future Work

In this paper, a virtual sensing application in the context of an open space office which has been regularly operating for several months has been considered. First, we systematically analysed various distance metrics that can be used to determine the best sensors on which to base a temperature prediction task. This also led to the design, by means of a genetic programming approach, of a combined metric, that can effectively be exploited for virtual sensing. Based on such findings, we proposed an original approach to sensor selection, and then conducted a thorough evaluation of several virtual sensing methodologies for the prediction of temperatures. Results showed that, within the considered setting, it is possible to achieve a satisfactory prediction performance using relatively simple models with a limited subset of predictor sensors in spite of the complex multi-parameter scenario.

As for future work, besides the directions already mentioned in the paper, we look forward to creating models to perform temperature interpolation and temperature trend simulation, and to compare their outcomes with those acquired from a specifically developed physical simulation of the room temperatures. Furthermore, on the basis of such simulation data, we will investigate techniques for the optimal arrangement of a limited number of sensors in a room. The proposed genetic programming-based distance metric is a first step in this direction. Finally, as a parallel research direction, we intend to incorporate some pieces of physical knowledge in the machine/deep learning models, according to a *grey-box* approach.

## Figures and Tables

**Table 1:** XGBoostRegressor parameters (rounded to the 5th decimal digit).

Parameter Name	max_depth	learning_rate	n_estimators	reg_alpha	reg_lambda	gamma	subsample	colsample_ bytree	min_child _weight
Value	16	0.015	350	78.87396	0.50044	5.95353	0.66425	0.65694	1.0

## Data Availability

Data available in a publicly accessible repository https://github.com/dslab-uniud, accessed on 7 April 2021.
